# First evidence of the presence of genotype-1 of Japanese encephalitis virus in *Culex gelidus* in Indonesia

**DOI:** 10.1186/s13071-018-3285-7

**Published:** 2019-01-08

**Authors:** Triwibowo Ambar Garjito, Mega Tyas Prihatin, Lulus Susanti, Dhian Prastowo, Siti Rofiatus Sa’adah, Yulian Taviv, Tri Baskoro Tunggul Satoto, Joko Waluyo, Sylvie Manguin, Roger Frutos

**Affiliations:** 1 Institute for Vector and Reservoir Control Research and Development (NIHRD-MoH), Salatiga, Indonesia; 2 Université de Montpellier, Montpellier, France; 3 HydroSciences Montpellier (HSM), Institut de Recherche pour le Développement (IRD), CNRS, Université de Montpellier, Montpellier, France; 4 Health Research and Development unit Baturaja, Baturaja, South Sumatra Indonesia; 5 Department of Parasitology, Faculty of Medicine, Gadjah Mada University, Yogyakarta, Indonesia; 6 CIRAD, Intertryp, Montpellier, France; 7 IES, Université de Montpellier-CNRS, Montpellier, France

**Keywords:** Japanese encephalitis, Genotype I, Indonesia, *Culex gelidus*

## Abstract

**Background:**

Japanese encephalitis has become a public health threat in Indonesia. Three genotypes have been recorded in Indonesia, i.e. genotype II (GII), genotype III (GIII) and genotype IV (GIV). Genotype I (GI) and genotype V (GV) have never been reported in Indonesia.

**Results:**

A Japanese encephalitis virus (JEV) belonging to the genotype I-a (GI-a) has been isolated for the first time from a *Culex gelidus* mosquito in the Province of Jambi, Indonesia. This virus is related to a 1983 isolate from Thailand whereas the infected *Cx. gelidus* mosquito belonged to a Chinese haplotype.

**Conclusions:**

Surveillance of JEV and mosquito dissemination is recommended.

## Background

The Japanese encephalitis virus (JEV) is a mosquito-borne flavivirus that has become a public health threat in Asia, including Indonesia. JEV is transmitted to humans through mosquito bites, especially of *Culex* species, from amplifier animals such as pigs. JEV can cause severe central nervous system disorders with high mortality or permanent neurological sequelae [1].

In Indonesia, JEV was first isolated from mosquitoes in West Java in 1972. Since then, encephalitis cases have been reported in several hospitals and currently Japanese encephalitis (JE) has become widespread and endemic across 32 out of 34 Indonesian provinces [2]. JEV originated from the IndoMalayan region and further evolved into five genotypes. Until now, only three genotypes have been recorded in Indonesia, i.e. genotype II (GII), genotype III (GIII) and genotype IV (GIV). Furthermore, GIV has only been described in mosquitoes in Indonesia [3]. Although JEV originated from the Indo-Malaysia region about 1695 years ago, genotype I (GI) most likely originated in Thailand for clade GI-a and Vietnam for clade GI-b about 193 years ago and has never been reported in Indonesia [3, 4]. Genotype I is associated with human encephalitis in China, Japan, India, Korea, Taiwan, Thailand and Vietnam [4]. GI JEV is an epidemic genotype with equal virulence as GIII JEV, the genotype most frequently associated with outbreaks in Asia [5].

## Methods

**Fig. 1:**
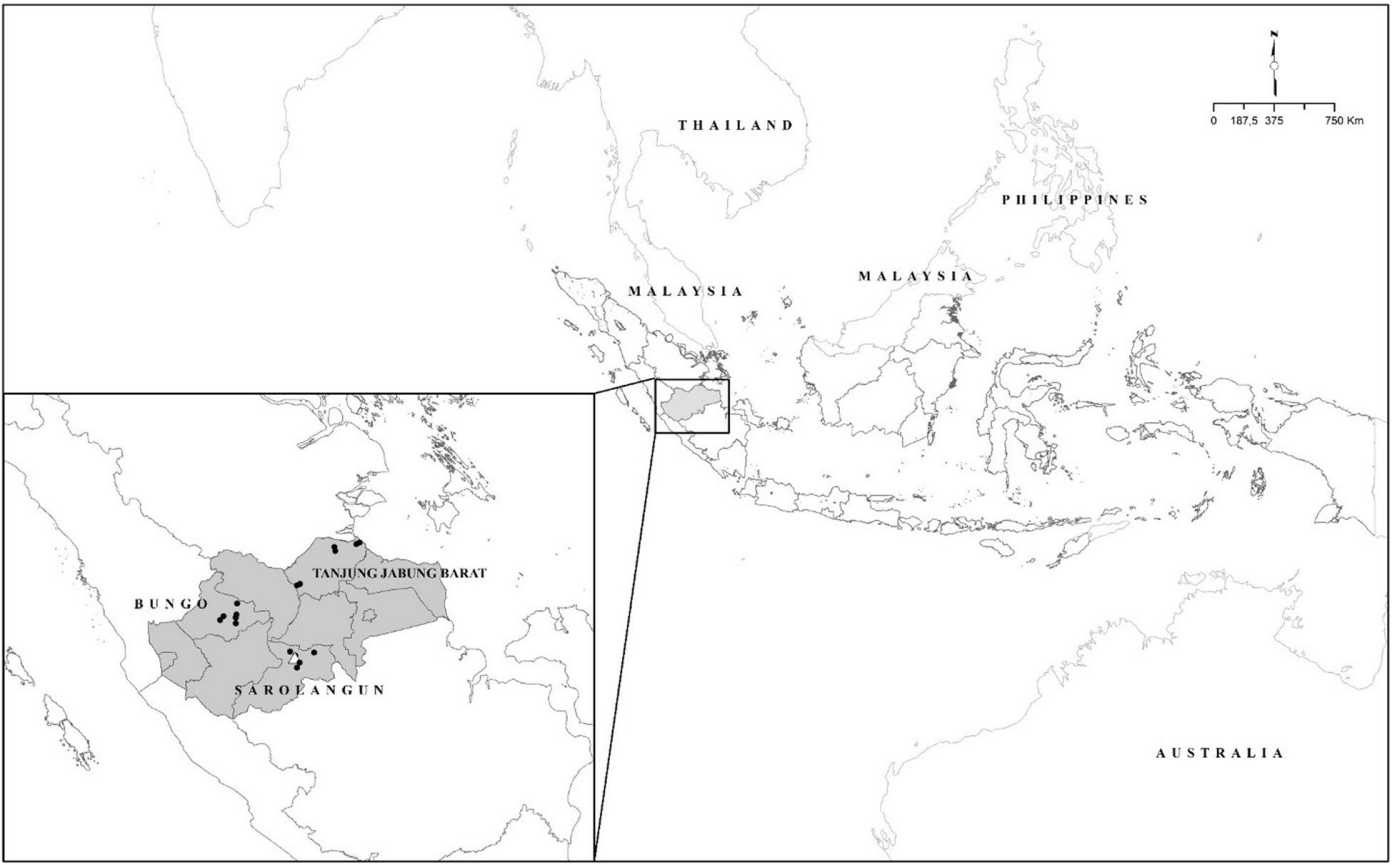
Map of Indonesia and of the province of Jambi. The Bungo, Tanjung Jabung Barat and Sarolangun districts where the sampling was conducted are located on the map. Black dots represent the sampling locations in each district (6 per district)

**Fig. 2:**
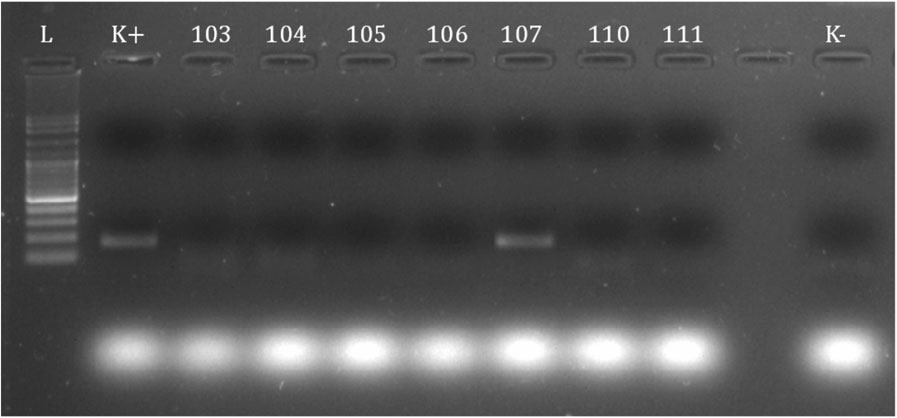
Electrophoretic analysis of JEV NS3 PCR products. Lane K+, positive control; Lane K-, negative control; Lane 107, Sample 107 (JE/mosq/jambi107/2017)

Jambi, Sumatra, is a province confirmed as a JE endemic area. Entomological investigation was conducted at six sentinel sites in the Bungo, Tanjung Jabung Barat and Sarolangun Districts from May to June 2017 (Fig. 1). Mosquito collections were conducted using human landing, direct collection around cattle and animal baited trap collection. Sampling was conducted from 18:00 h to 6:00 h at every sentinel site. After identification, mosquitoes were sorted according to locality and date, and stored in RNAlater (Ambion-Thermo Fischer Scientific, Waltham, USA) at -80 °C until further analysis. The excised head and thorax of each mosquito were homogenized in a sterile homogenizer, RNA was extracted by silica-based methods (RNA-easy minikit, Qiagen, Hilden, Germany) and sample aliquots were pooled together by groups of 25. JEV detection was carried out by one step RT-PCR on the NS3 gene [6] using the consensus primers FP (5'-AGA GCG GGG AAA AAG GTC AT-3') and RP (5'-TTT CAC GCT CTT TCT ACA GT-3'). The PCR reaction was performed as previously described [7]. The primers corresponded to a 162-bp product (Fig. 2). JEV genotyping from positive samples was performed using the envelope (E) gene sequence. The E gene was amplified using the primers JEV-Ef (5'-TGY TGG TCG CTC CGG CTT A-3') and JEV-Er (5'-AAG ATG CCA CTT CCA CAY CTC-3') [7] using Superscript III one-step RT-PCR with platinum *Taq*DNA polymerase (Invitrogen, Life Technologies, Carlsbad, USA). For amplification of the JEV gene, initial denaturation was carried out at 93 °C for 30 s followed by 40 cycles of 94 °C for 15 s, 53 °C for 30 s and 68 °C for 1 min 30 s, with a final extention step at 68 °C for 5 min. Products were electrophoresed in 2% agarose gel and vizualized by SYBR safe DNA gel staining (Invitrogen, Life Technologies).

The amplification products were then purified using Illustra ExoProSTar (GE Healthcare Life Sciences, Tokyo, Japan). Sequencing of the amplified gene E was performed using the primers JEV-Ef and JEV-Er mentioned above and ABI BigDye terminator Cycle Sequencing Kit v.3.1 (Applied Biosystems, Austin, USA). Prior to sequencing, a PCR product purification step was performed using BigDye Xterminator Purification Kit (Applied Biosystems). Sequence data were obtained through the Sanger method using an automatic DNA sequencer (Applied Biosystems 3500 Genetic Analyzer) and analyzed using the Sequencing Analysis 6 program (Applied Biosystems).

The phylogenetic tree was built using the maximum likelihood method with Tamura Nei (TN93) as evolutionary model. Node bootstraps were calculated with 2000 replicates. Sequences were compared to 16 JEV reference sequences from GenBank comprising 6 GI-b, 2 GI-a, 2 GII, 3 GIII, 2 GIV, 1 GV and 2 MVEV (Murray Valley encephalitis virus) sequences used as outgroups. The mosquito in which the JEV was detected was genotyped using the cytochrome *c* oxidase subunit 1 gene (*cox*1) as a target. The *cox*1 gene was amplified using the primers CIN2087 (5'-AAT TTC GGT CAG TTA ATA ATA TAG-3') and TYJ-1460 (5'-TAC AAT TTA TCG CCT AAA CTT CAG CC-3') as previously described [8].

## Results and discussion

**Fig. 3:**
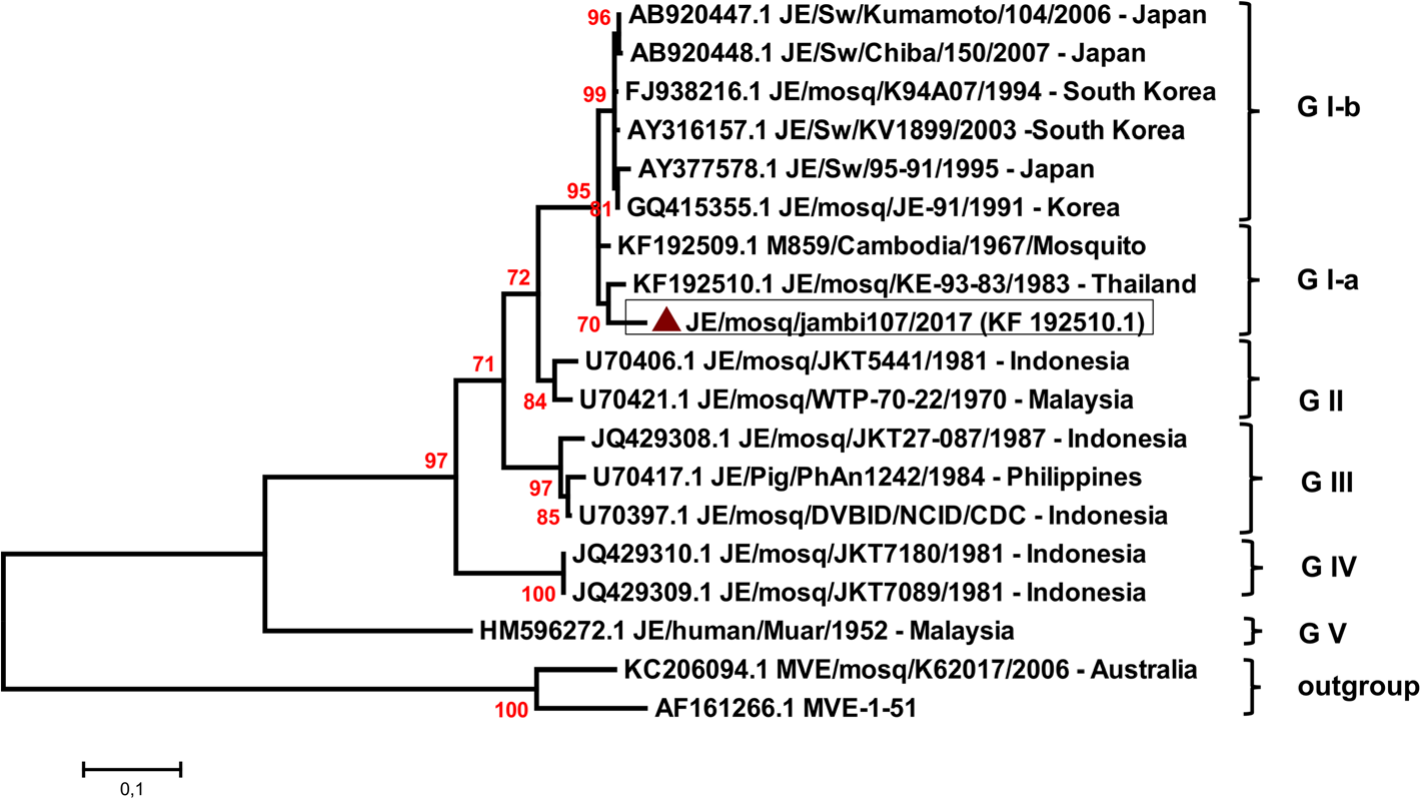
Phylogenetic tree positioning JE/mosq/jambi107/2017 in comparison to 16 JEV reference strains. Reference JEV sequences were obtained from GenBank. Reference sequences are identified by their accession number. JE/mosq/jambi107/2017 (KF192510.1) is shown in a box and marked by a red triangle. The sequence from this study is marked by a red triangle. The phylogenetic analysis was performed with the maximum likelihood method using the Tamura-Nei model (TN93) as evolutionary model. Node bootstraps were calculated with 2000 replicates. Bootstrap values < 70 are not shown. The tree was rooted with 2 Murray Valley encephalitis virus (MVEV) sequences as outgroups. The scale-bar indicates the number of nucleotide subtitutions per site

A total of 1485 *Culex* mosquitoes were collected and analyzed. These mosquitoes belonged to five different species: *Culex gelidus*, *Culex quinquefasciatus*, *Culex tritaeniorhynchus*, *Culex vishnui* and *Culex fuscocephalus* (Table 1). The species displaying the highest prevalence were *Cx. gelidus* and *Cx. quinquefasciatus*. JEV was detected in only one *Cx. gelidus* mosquito. Positive detection of JEV was confirmed by sequencing and blast analysis. The gene E sequence from JE/mosq/Jambi107/2017 was deposited in GenBank under the accession number MK032889. The gene E phylogenetic analysis (Fig. 3) indicated that JE/mosq/Jambi107/2017 belonged to the clade GI-a of JEV and was closely related to a genotype I-a isolate from Thailand (GenBank: KF192510.1). The GI-a clade of genotype I was described until now only in Thailand and Cambodia. The genotype I of JEV is found only from 10°N up to 35°N with two separate clades [3, 4]. The clade GI-a is found in Thailand and Cambodia from where one case was introduced to Australia [3, 4]. The clade GI-b is found in Vietnam, China, Taiwan, Korea, India and Japan [3, 4].

**Table 1:** *Culex* mosquitoes captured in the Province of Jambi, Indonesia

Species	No. of pools	No. of samples
*Cx. fuscocephalus*	2	10
*Cx. gelidus*	34	850
*Cx. quinquefasciatus*	23	500
*Cx. tritaeniorhynchus*	2	50
*Cx. vishnui*	3	75
Total	64	1485

To our knowledge, this is the first detection of GI JEV in Indonesia. A replacement of GIII by GI was reported throughout Asia and Australia since 1979 [9]. A similar phenomenon could be underway in Indonesia and should be investigated more thoroughly. The mode of introduction of GI in several countries has not been clearly established but a narrower vector host range and a higher replication capacity of GI in mosquitoes has been described [3]. The *cox*1 haplotype of the *Cx. gelidus* mosquito infected with JE/mosq/Jambi107/2017 (GenBank: MK045308) was found to be very close to a haplotype previously described in China (GenBank: MF179173). Blast results for the two best hits were as follows: GenBank: MF179173, maximum score 861, total score 861, total coverage 100%, E value 0.0, identity 97%; GenBank: MF179172, maximum score 856, total score 856, total coverage 100%, E value 0.0, identity 97%. *Culex gelidus* is a good vector of JEV with an invasive capacity and a potential for being transported over long distance by boats, planes or road transportation [10].

## Conclusions

The role of specific mosquito populations in the introduction and dissemination of GI JEV through commercial routes should be investigated. GI, which is currently replacing GIII in Asia, could not be detected in cerebrospinal fluid by JEV-specific IgM antibodies raised against GIII JEV [3]. There is thus a risk of misdiagnosis in the presence of GI. Furthermore, all vaccines currently available against JEV are derived from GIII JEV and several studies have reported human confirmed cases with GI JEV infection in areas where effective JEV vaccination programs exist [11, 12]. There is thus, in addition to misdiagnosis, a risk of lack of efficient protection associated with the extension of GI. Further studies and strengthened JE surveillance should be implemented to assess the distribution of GI JEV in Indonesia and health authorities must be alerted in order to address potential risks to public health.

## Abbreviations

JEV: Japanese encephalitis virus
G: genotype
*cox*1: cytochrome *c* oxidase subunit 1
MVEV: Murray Valley encephalitis virus
